# The intersection of school corporal punishment and associated factors: Baseline results from a randomized controlled trial in Pakistan

**DOI:** 10.1371/journal.pone.0206032

**Published:** 2018-10-24

**Authors:** Hussain Maqbool Ahmed Khuwaja, Rozina Karmaliani, Judith McFarlane, Rozina Somani, Saleema Gulzar, Tazeen Saeed Ali, Zahra Shaheen Premani, Esnat D. Chirwa, Rachel Jewkes

**Affiliations:** 1 School of Nursing and Midwifery, Aga Khan University, Karachi, Pakistan; 2 Texas Woman’s University, Houston, Texas, United States of America; 3 South African Medical Research Council, Pretoria, South Africa; Wayne State University, UNITED STATES

## Abstract

Violence against youth is a global issue; one form of youth victimization is school corporal punishment. We use baseline assessments from a cluster randomized controlled trial to examine the prevalence of school corporal punishment, by gender, and the relationship to levels of peer violence at school, parent corporal punishment, youth food security and youth academic performance and school attendance in Pakistan. Forty homogenous public schools in the urban city of Hyderabad, Pakistan were chosen for randomization into the trial evaluating a youth violence prevention intervention. 1752 6^th^ graders, age 11–14 years, were selected as the target population. Since schools are segregated by gender in Pakistan, data are from interviews in 20 boys’ schools and 20 girls’ schools. Overall, 91.4% of boys and 60.9% of girls reported corporal punishment at school in the previous 4 weeks and 60.3% of boys had been physically punished at home in the past 4 weeks compared to 37.1% of girls. Structural equation modeling revealed one direct pathway for both boys and girls from food insecurity to corporal punishment at school while indirect pathways were mediated by depression, the number of days missed from school and school performance and for boys also by engagement in peer violence. Exposure to corporal punishment in school and from parents differs by gender, but in both boys and girls poverty in the form of food insecurity was an important risk factor, with the result that poorer children are victimized more by adults.

## Introduction

Corporal punishment is defined as the use of physical force with the intent to cause pain as a mode of punishment, modification of behavior or preventing the negative behavior [1]. Corporal punishment in the school and home settings is quite common. A systematic review of school corporal punishment found that the occurrence varied from 13% of students in Kazakhstan to fully 97% of students in Cameroon [2]. Higher reports of school corporal punishment consistently come from resource-poor countries [3, 4], most likely due to legal sanctions against corporal punishment and lower parental support for its use in mid to high resourced countries, such as few states of North America and Europe. According to the UNICEF report of 2010, where data was collected from 35 middle and high income countries, three out of four children experience violent discipline at home [5].

Surveys document that corporal punishment can have negative impacts on child behavior [6]. For example, children who are spanked at home are more likely to participate in violent acts and demonstrate higher levels of aggression throughout adolescence [7, 8].

### Contributing factors to corporal punishment

Corporal punishment is prevalent and occurs because of inter-related social, cultural and educational factors [9], and can be due to a power imbalance, such as power differentials between different ethnic groups, castes and classes, and oppression of ethnic minorities [10]. For example children in Turkey who report corporal punishment at school frequently belong to lower social-economic groups [11]. A common reason to justify corporal punishment is discipline. Therefore school policies may justify corporal punishment as needed to teach children how to refrain from unwanted behaviors and maintain school decorum [12]. Once corporal punishment is justified and integrated into the culture of the school, the practice becomes accepted and viewed as a ‘necessary’ part of teaching.

Teacher attitudes and their socio economic status also serve as contributing factors. A study conducted in Turkey revealed that when the teacher is overwhelmed with personal problems, he or she is less likely to manage behavior problems in a non-violent manner and more likely to use corporal punishment [11]. A study conducted in Nigeria by Ogbe (2015) to evaluate parents and teacher’s perception of using corporal punishment in schools revealed that both parents and teachers feel that using corporal punishment is the right way to mold children’s behavior and if you ‘spare the child, you spoil the child’. The study found no gender differences among urban and rural parents or teachers on the agreed justifications for using corporal punishment. In fact, the parents surveyed by Ogbe (2015) recommended working towards legislation to allow corporal punishment at schools and protecting teachers from retribution when applying corporal punishment.

### Outcomes of corporal punishment

Research reveals that, students who are victims of corporal punishment are less motivated towards their class room activities. The youth lack confidence, hesitate to become involved in group discussions, and are less motivated towards studies [13]. The UNICEF’s Asian Report (2001) describes how corporal punishment may cause severe physical injuries and disabilities among children and even result in child death. For example, eardrum damage can result from a forceful slap to the head by parents or teachers. Corporal punishment can affect the mental health of children and result in youth feeling helpless, hopeless, worthless, depressed, and inadequate [14]. Corporal punishment is a primary contributor to child abuse and promotes violent behavior between children [15]. A study indicates that physical punishment of children increases antisocial attitudes among children and decreases children’s capacity to learn the behavior that the corporal punishment was intended to promote (Gershoff, 2008). Corporal punishment forms part of a cycle of violence within the family and school settings which can become engrained and justified as the ‘best’ strategy for shaping youth behavior [16].

Corporal punishment at school is also associated with school dropout. A study conducted in primary schools in Pakistan identified that corporal punishment as the primary reason for children leaving school [17]. Children who are victims of corporal punishment are commonly perpetrators of violence against family and society [18]. Another study conducted in Pakistan linked school corporal punishment to poor academic performance and lack of youth confidence along with anxiety and fear [19].

Clearly, corporal punishment by teachers and parents is pervasive and detrimental to youth development and academic performance. Over the last decade there has been a growing recognition internationally of the importance of children’s experience of violence, of various types, and the need to prevent it. What Works To prevent Violence Against Women and Girls? Global Program is funded by the United Kingdom’s Department for International Development (DFID) [20] and evaluating interventions in 14 countries to prevent violence against women and girls. One of the evaluations is located in urban public schools in Pakistan and seeks to empower children to prevent violence against youth, including school and parent corporal punishment, through a two-year programme of play-based activities called “The Positive Child and Youth Development program of the Right to Play” (2017) Pakistan office [21]. Additionally, a detailed description of the Right to Play intervention as evaluated in this research is presented elsewhere [22].

If violence against youth is to be prevented and meaningful interventions applied, contextual factors must be examined and understood. Using the baseline data of this 2-year RCT, this paper describes the prevalence of school corporal punishment, by gender, and associations with youth peer victimization and perpetration, corporal punishment at home and food security, and finally, youth academic performance and attendance.

## Methods

The data are from the baseline of a cluster randomized controlled trial. The details of the intervention as well as methods of the 2-year cluster randomized controlled trial are described in detail elsewhere [22]. The clusters are forty fairly homogenous schools in the urban city of Hyderabad, which is located in the southern province of Sindh, about 100 miles from the mega city of Karachi. All the schools selected for this study were single sex public schools. Half the schools were girls’ schools and half boys’ schools [22].

The study population was youth in the 6^th^ grade, mostly 11–14 years of age. The inclusion criteria for children were that they should be students in grade 6 in selected schools, obtain consent for the study from their parents, and agree to participate themselves. The youth needed to read the national language of Pakistan Urdu or the provincial language Sindhi. The questionnaires were self-completed, with children divided into groups of 3–4 with an interviewer. Proceeding item by item, the interviewer read the question and the children completed the questionnaire. In small schools we invited the whole of Grade 6 to participate, but if the grade was large if was often divided into 2 or 3 sections and then we invited just one section. The number of children recruited per school ranged from 20–65.

Following Internal Review Board Approval and completion of informed consent procedures, consisting of parental written consent and child assent, pilot testing of the instruments with 124 youth in the 6^th^ grade in an adjacent school district was completed. Adjustments were made to streamline and shorten the data collection process. Data collection for the 40 schools was completed over a 60-day period in 2015.

For the 40 schools, we sent home a total of 2,486 parental consent forms and received 1,858 affirmed parent consents for a return rate of 75%. Of the 1,858 forms signed and returned by the parents, 1,767 children assented for a rate of 95%. In general parents of girls consented more often than parents of boy, 79% compared to 70% respectively. A total of 1,752 youth (822 boys and 930 girls) completed questionnaires and were entered into an SPSS database.

Corporal punishment at school is a 6-item investigator derived scale that asked the frequency, (i.e., never, once, few times (2–3), or many times (4 or more) with which youth were punished by a teacher within the last 4 weeks. Cronbach’s Alpha was 0.757 for both boys and girls [22]. Items include slapped, hit or beaten as well as made to run, kneel, or stand. These are common forms of punishment used in Sindh schools. A dichotomous outcome was derived from the 6 items, with 0 representing learners that had never experienced any of the 6 acts and 1 representing learners that had experienced at least one of the 6 acts.

Two questions asked about parental physical punishment frequency (i.e., never, once, few times (2–3), or many times (4 or more) and severity at home during the past 4 weeks.

Peer Victimization Scale of 16-items was used with 4 subscales, each with 4 questions, assessing physical and verbal victimization, social manipulation, and property attacks [23]. Respondents were asked over the last 4 weeks, how often (i.e., never, once, a few times (2–3) or many times (4 or more) an event happened to them (i.e. victimization). Scale scores were computed by summing item responses (range of 0 to 48). Higher scores reflect more victimization. A Peer Perpetration Scale was developed for the study based on the peer victimization scale. We asked the same 16-items of the peer victimization scale with the wording adjusted to measure perpetration [23]. It has the same 4 subscales and scoring. Cronbach’s alpha was 0.86 for both girls and boys for victimization and Cronbach’s alpha was 0.86 for both girls and 0.89 for boys for perpetration.

Peer victimization and perpetration were categorized using thresholds suggested by the U.S. Centers for Disease Control and Prevention (CDC) guidelines [24]. These guidelines define a participant score on the Peer Victimization Scale or Peer Perpetration Scale of 0 to 1 as low violence and 2 or greater as high violence in the last 4 weeks.

The questionnaire also asked about food security, parent literacy, and home assets and amenities. A hunger score was created from two items that asked learners how often they had gone to school without breakfast in the last 4 weeks, and also how often in the last 4 weeks they had gone to sleep without dinner due to lack of food at home. The two items were measured on a 4-point scale, with 0 indicating ‘never’, and 3 indicating ‘all or most days’. Child academic performance was measured in a 7-item, investigator derived, set of questions that assessed the child’s self-report of performance in language, mathematics, science and social studies (i.e., below average, average, above average). The number of days missed from school in the preceding 4 weeks was tallied, and the reasons for the absences recorded.

Child’s Mental Health was assessed by Children’s Depression Inventory II (CDI-2): a 28-item self-report questionnaire to assess the severity of current or recent (last 2 weeks) depressive symptoms. The response options for each item are rated on a 3-point scale as: 0 (no symptom), 1 (probable or mild symptom), and 2 (definite, marked symptom). The range of scores is 0–56 with higher scores representing increased depressive symptoms. CDI-2 For this study, the raw scores for the CDI-2 were converted into t-scores based on age and gender attributes of the participants according to the specifications in the CDI-2 technical manual [25]. The T-scores range from ≤40 to ≥90.

### Data analysis

All analysis of this baseline study took into account the study design, which is a cluster randomized control design, with participants clustered within schools. Descriptive analyses were carried out on all potential explanatory variables and were summarized by sex of participant. These variables included family life characteristics, home violence experience, school attendance/performance, and peer victimization and perpetration of violence. Frequencies/means and percentages/standard deviations were used to summarize the variables.

We used logistic regression modeling to measure bivariable relationships between corporal punishment and factors associated with experience of corporal punishment, with standard errors estimated using the clustered robust method in order to account for clustering of participants by school.

We then fitted a path model for girls and boys using full information maximum likelihood estimation (FIML) to allow for modeling of missing data, corporal punishment measured as a latent construct of the 6 items. The final models were built based on theory and statistically meaningful modifications using backwards elimination to exclude endogenous variables that did not mediate any path (with significance set at the p < .05 level) from the exogenous variables to corporal punishment. All factors that were significantly associated with corporal punishment experience in the bivariate analysis were considered for the path model. We followed Anderson and Gerbing’s procedure for testing fit of a structural equation model, performing confirmatory factor analysis to assess the relationship between latent factors. Before adjusting standard errors for clustering of participants in schools, model fit was good (boys: RMSEA = 0.018, CFI = 0.993, TLI = 0.989; girls: RMSEA = 0.025, CFI = 0.991, TLI = 0.983). We tested the significance of mediation using the Sobel test, by looking at direct and indirect effects of the explanatory variables on corporal punishment experience.

All analyses were done in Stata 14.

## Results

The mean age of the boys participating was 12.5 years and of the girls was 12.3 years (Table 1). Food insecurity, measured through having missed breakfast or dinner in the past month due to lack of food, was captured in a hunger score, which was higher for boys then for girls. School attendance was irregular with boys having missed an average of 4.1 days school in the previous month and girls an average of 3.1 days. The last day missed from school was due to having to work at home in the cases of 24.5% of boys and 14.2%, whilst 7.7% of boys and 1.6% of girls had missed school due to the need to work to earn money. Self-reported school performance was higher for girls than boys. Boys reported slightly higher parental literacy than girls.

**Table 1:** Social and demographic characteristics of boys and girls in the sample.

	N	Boys		Girls	
		n	mean (sd) / %	n	mean (sd)/ %
Age of learner‡	1748		12.51 (1.5)		12.27 (1.38)
Hunger Index‡	1752		0.65 (1.09)		0.48 (0.97)
Number of days missed school‡	1740		4.07 (4.17)		3.14 (2.85)
Last day missed from school was due to working at home	325	195	24.5	130	14.2
Last day missed from school was due to working for money	76	61	7.7	15	1.6
School performance‡	1752		9.27 (1.81)		9.55 (1.77)
Parent literacy levels‡	1752		2.61 (1.41)		2.54 (1.41)
Peer victimisation/ perpetration					
None	249	55	6.7	194	20.9
Victimisation only	411	146	17.8	265	28.5
Any perpetration	1092	621	75.5	471	50.6
Child attitudes to physical punishment‡	1752		9.33 (3.22)		9.93 (3.39)
Experienced physical punishment at home	840	495	60.3	345	37.1

^‡^: summaries are means and standard deviations.

sd = standard deviation

Boys had a greater involvement in peer violence than did girls. Only 6.7% of boys and 20.9% of girls had no experience of violence as a victim or perpetrator in the previous 4 weeks. 75.5% of boys had perpetrated violence and 50.6% of girls, and many of these had also experienced it as victims. Girls reported lower acceptability of children being beaten for punishment than boys, mean scores 9.93 for girls and 9.33 for boys. Overall 60.3% of boys had been physically punished at home in the past 4 weeks and 37.1% of girls.

Table 2 shows that 91.4% of boys and 60.9% of girls had experienced corporal punishment at school in the previous 4 weeks. Three quarters of boys and one third of girls had been slapped, beaten, hit or otherwise physically punished and especially for boys, this was often on multiple occasions. 47.4% of boys and 10.5% of girls had had their ear twisted by a teacher. Being made to stand on a bench was the only form of punishment which did not show a gender difference, with 42.2% of boys and 36.9% of girls reporting having done this. Boys were often asked to run around outside (16.9% v. 3.9% for girls), and 42.9% of boys had been made to kneel outside compared to 7% of girls. 68.7% of boys and 19.1% of girls were hit with a stick by a teacher in the last 4 weeks.

**Table 2:** Prevalence of experience of acts of corporal punishment in the previous four weeks.

	Boys		Girls		
	n	%	n	%	p value
Were you slapped, hit or beaten or otherwise physically punished by a teacher?					
Never	219	26.6	623	67	<0.001
Once	287	34.9	238	25.6	
Few times	170	20.7	52	5.6	
Many times	146	17.8	17	1.8	
Did a teacher twist your ear?					
Never	432	52.6	832	89.5	<0.001
Once	229	27.9	73	7.8	
Few times	99	12	20	2.2	
Many times	62	7.5	5	0.5	
Did a teacher make you stand on a bench?					
Never	476	57.9	587	63.1	0.132
Once	211	25.7	237	25.5	
Few times	81	9.9	66	7.1	
Many times	54	6.6	40	4.3	
Did a teacher make you run around as punishment?					
Never	683	83.1	894	96.1	<0.001
Once	80	9.7	21	2.3	
Few times	42	5.1	14	1.5	
Many times	17	2.1	1	0.1	
Did a teacher make you kneel down in class or outside?					
Never	470	57.2	865	93	<0.001
Once	203	24.7	49	5.3	
Few times	91	11.1	14	1.5	
Many times	58	7.1	2	0.2	
In past 4 weeks, did a teacher hit you with a stick?					
Never	257	31.3	753	81	<0.001
Once	247	30	121	13	
Few times	154	18.7	47	5.1	
Many times	164	20	9	1	
Experienced any physical punishment					
No	71	8.6	364	39.1	<0.001
Yes	751	91.4	566	60.9	

Bi-variable associations between a range of social, home and school factors and experience of corporal punishment are shown in Table 3. Among boys, experience of corporal punishment at school was more likely if school performance was poorer, if the boys had been beaten at home and if they had been involved in peer violence either just as a victim, or as a perpetrator. It was not associated with age, food insecurity, parental literacy or days missed from school. Among girls, corporal punishment at school was associated with missing more days from school and having poorer school performance. It was also associated with being beaten at home and engaging in peer violence again both as a perpetrator, but also when only a victim.

**Table 3:** Bivariable associations between experience of corporal punishment by boys and girls and social and demographic characteristics.

	BOYS								GIRLS							
	Experienced corporal punishment								Experienced corporal punishment							
	Yes		No						Yes		No					
	mean/n	sd/%	mean/n	sd/%	OR	LCL	UCL	p value	mean/n	sd/%	mean/n	sd/%	OR	LCL	UCL	p value
Age of learner‡	12.5	1.47	12.68	1.77	0.93	0.77	1.10	0.42	12.25	1.36	12.3	1.42	0.98	0.91	1.05	0.54
Hunger Index‡	0.66	1.08	0.52	1.12	1.14	0.85	1.54	0.31	0.55	1.02	0.37	0.87	1.25	0.99	1.57	0.06
Number of days missed school‡	4.04	4.11	4.31	4.73	0.99	0.94	1.03	0.59	3.37	2.81	2.79	2.87	1.08	1.03	1.13	0.02
School performance‡	9.21	1.81	9.97	1.64	0.77	0.68	0.87	0.03	9.36	1.86	9.85	1.58	0.85	0.80	0.90	0.02
Parent literacy levels‡	2.62	1.39	2.51	1.59	1.06	0.96	1.17	0.31	2.55	1.39	2.53	1.43	1.01	0.93	1.10	0.77
Child attitude to physical punishment	9.36	3.21	9.01	3.34	1.04	0.95	1.13	0.44	9.98	3.26	9.86	3.58	1.01	0.98	1.04	0.479
Experienced physical punishment at home	483	64.4	12	16.9	8.89	4.10	19.28	<0.001	272	48.1	73	20.1	8.89	4.10	19.28	<0.001
Peer victimisation and perpetration																
None	34	4.5	21	29.6					58	10.2	136	37.4				
Victimisation only	118	15.7	28	39.4	2.60	1.86	3.65	<0.001	138	24.4	127	34.9	2.60	1.86	3.65	<0.001
Any perpetration	599	79.8	22	31	16.82	11.12	25.43		370	65.4	101	27.8	16.82	11.12	25.43	
Last day missed from school was due to working at home	181	25	14	20	1.33	0.78	2.26	0.31	86	15.6	44	12.1	1.33	0.78	2.26	0.16
Last day missed from school was due to working for money	56	7.7	5	7	1.10	0.47	2.57	0.82	9	1.6	6	1.7	1.10	0.47	2.57	0.98

^‡^: comparison done on means;

sd = standard deviation; OR = Odds ratio; LCL = lower confidence limit; UCL = Upper Confidence limit

**Fig 1:**
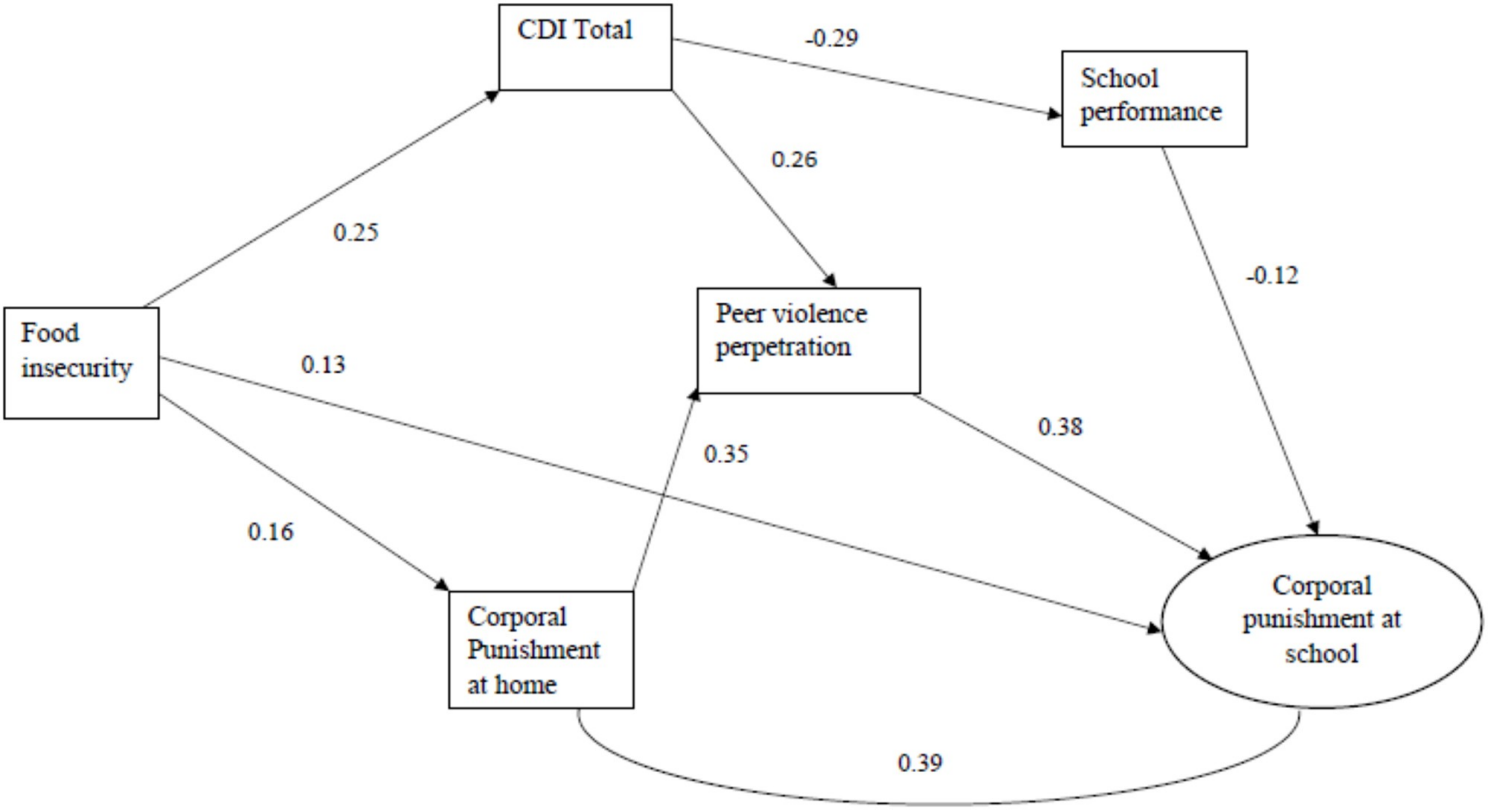
Final structural equation model of corporal punishment at school experienced by boys.

**Fig 2:**
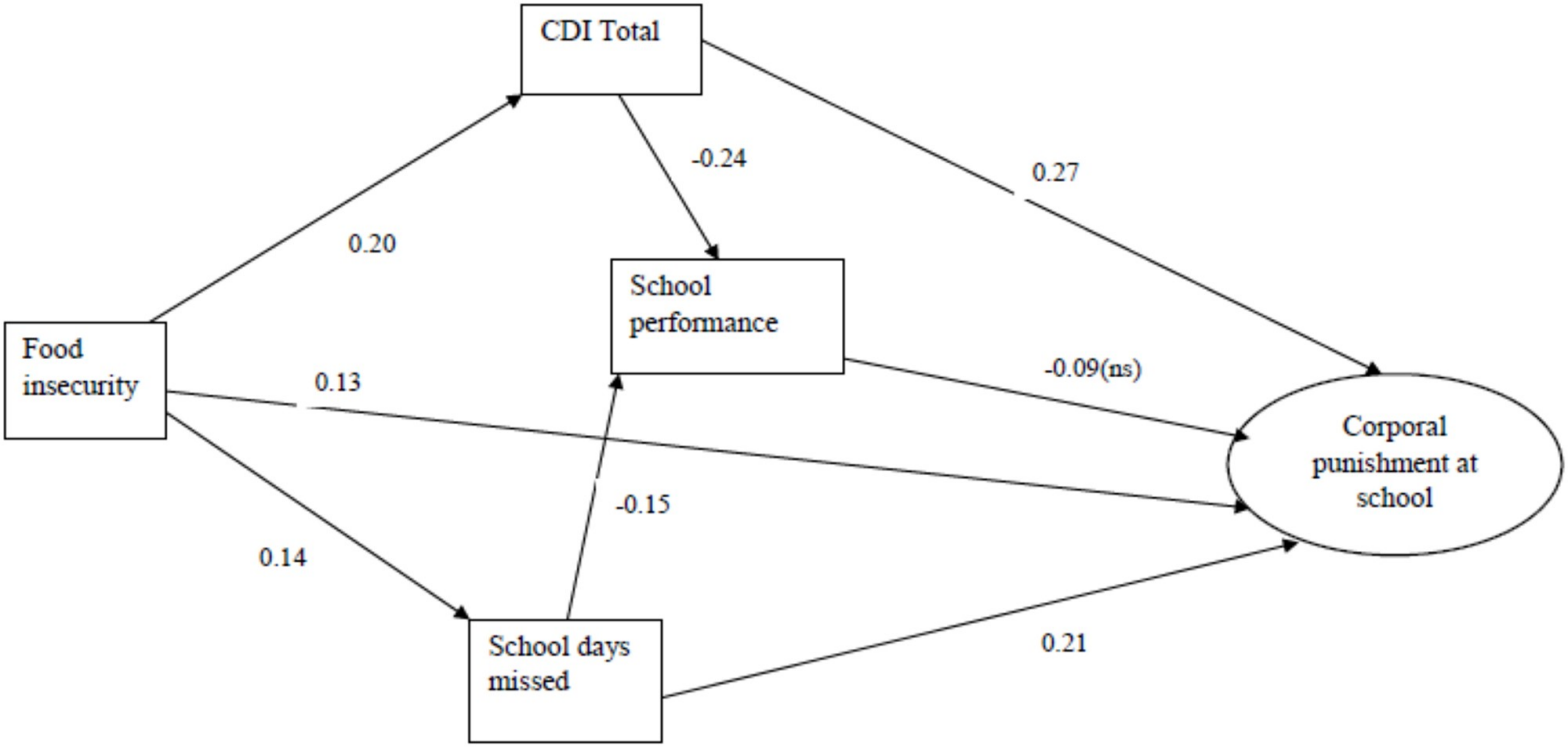
Final structural equation model of corporal punishment at school experienced by girls.

The structural equation models for experience of corporal punishment among boys and girls are presented in Figs 1 and 2 and Table 4. For boys, the structural model has one direct pathway from the hunger index (food insecurity) to corporal punishment at school. There are five indirect pathways. One is mediated by depression and poor school performance. One is mediated by depression and peer violence. There is strong correlation between experiencing corporal punishment at home and experiencing corporal punishment at school. One is mediated by corporal punishment at home and peer violence. Fifth pathway is mediated by corporal punishment at home and depression and poor school performance or peer violence. Standardized coefficients in Table 4 show strong direct effect of peer violence perpetration on experience of corporal punishment at school (β = 0.38).

**Table 4:** Results of the structural equation model analysis.

	BOYS				β	GIRLS				β
	Coef.	LCL	UCL	p value		Coef.	LCL	UCL	p value	
**Direct effects**										
Hunger score->school days missed						0.42	0.12	0.73	<0.001	0.14
School days missed --> School performance						-0.10	-0.13	-0.06	<0.001	-0.15
Hunger score -> CDI-2 Total	2.19	1.59	2.79	<0.001	0.25	1.93	1.00	2.85	<0.001	0.20
Hunger score -> Corporal punishment at home	0.18	0.10	0.27	<0.001	0.16					
Corporal punishment at home ---> CDI-2 Total	1.25	0.61	1.89	<0.001	0.16					
CDI-2 Total-->School performance	-0.05	-0.04	-0.29	<0.001	-0.29	-0.05	-0.03	-0.24	<0.001	-0.24
CDI-2 Total--> Peer violence	0.20	0.15	0.25	<0.001	0.26					
Corporal punishment at home ---> Peer violence	2.09	1.56	2.62	<0.001	0.35					
School performance ---> Corporal punishment at school	-0.04	-0.07	-0.02	0.001	-0.12	-0.02	-0.04	0.01	0.169	-0.09
Peer violence ---> Corporal punishment at school	0.03	0.02	0.04	<0.001	0.38					
Hunger score -> Corporal punishment at school	0.08	0.02	0.13	0.008	0.13	0.05	0.01	0.08	0.007	0.13
CDI-2 Total ---> Corporal punishment at school						0.02	0.01	0.03	<0.001	0.27
School days missed---> Corporal punishment at school						0.03	0.01	0.05	0.042	0.21
Covariance(Corporal punishment at home and Corporal Punishment at school)	0.39	0.29	0.50	<0.001						
**Disturbance Variance**										
School performance	3.00	2.61	3.44			2.85	2.43	3.36		
Corporal Punishment at home	1.59	1.37	1.84							
Corporal Punishment at school	0.31	0.25	0.39			0.11	0.07	0.17		
CDI-2 Total	29.0	25.8	32.5			28.9	25.8	32.5		
Peer violence	43.7	35.9	53.3							
Days missed from school						7.92	6.76	9.30		
**Equation level goodness of fit**	R^2^	mc	mc2			R^2^	mc	mc2		
School days missed						0.02	0.14	0.02		
School performance	0.08	0.29	0.08			0.08	0.29	0.08		
Corporal Punishment at school	0.29	0.55	0.29			0.18	0.43	0.18		
Peer violence	0.23	0.48	0.23							
Corporal punishment at home	0.03	0.16	0.03							
CDI-2 Total	0.10	0.32	0.10			0.04	0.20	0.04		
**Overall goodness of fit**	CFI	TLI	RMSEA			CFI	TLI	RMSEA		
	0.993	0.989	0.018			0.991	0.983	0.025		
Overall R^2^	0.092					0.077				

Note: mc2 = R-squares is the Bentler-Raykov squared multiple correlation coefficient

mc = correlation coefficient between depvar and its prediction

β = standardized coefficient of the direct effects. LCL = Lower confidence limit; UCL = Upper Confidence Limit

For girls, there are three pathways from the hunger index to experience of corporal punishment at school. There is a direct pathway, such that more food insecurity is associated with a greater likelihood of being punished. An indirect pathway is mediated through the number of days missed from school, and a second indirect pathway mediated by depression and a third pathway which is mediated by depression, poor school performance and days missed from school. Although the direct effect of school performance on experience of corporal punishment at school is not statistically significant, the direction of the relationship indicate poor performance is associated with corporal punishment experience (β = -0.09). The standardized coefficients in Table 4 show that depression and school days missed have strong direct effect on corporal punishment experience (β = 0.27, & β = 0.21 respectively). The standardized and unstandardized coefficients, p values and direct, as well as R2 are presented in Table 4.

## Discussion

Among this sample of boys and girls attending the 6^th^ grade in public schools in Pakistan, there were several key differences between gender groups whereby boys reported being punished more than the girls both at school and home. These findings are in agreement with previous research from Pakistan where 47.5% boys and 32.1% of girls between 12 to 14 years age experience physical punishment by teachers [26]. Research from other countries has also shown the same pattern of gender differences in the use of corporal punishment, for example a study in Tanzania also found school corporal punishment was significantly more common for boys than girls [4].

In our study boys who reported higher levels of parent corporal punishment also reported higher school corporal punishment, Although our research cannot establish temporal sequencing, a meta-analysis of 27 studies, reports every study found physical punishment at home was associated with more, not less child aggression [27]. Worldwide, greater use of corporal punishment in the home is associated with more child behavior problems, irrespective of cultural and ethnic differences [8]. Research from Egypt shows that anti-social behavior of youth is punished by parents through corporal punishment and this same behavior is punished by teachers [28]. Aggressions is closely associated with depression in adolescents [29], and we have shown elsewhere that corporal punishment at home is strongly associated with depression, as is peer violence engagement in girls [30], and our structural model shows a pathway from corporal punishment at home to corporal punishment at school mediated by engagement in peer violence. This indicates that primary prevention of child aggression should involve stopping corporal punishment at home and this would reduce corporal punishment in school. It has also been observed that boys look up to their parents especially their fathers as role models. If parents are fighting with each other or with others to resolve the conflict, these boys learn this behavior and practice at school and community level. Their parents and teachers then try to ‘fix’ their behavior by corporal punishment [31].

The Sustainable Development Goals aim to end all forms of hunger and malnutrition by 2030, ensuring everyone has access to adequate and nutritive food [32]. The priority of Millennium Development Goals was also to reduce global hunger to 50% but unfortunately the numbers of malnourished people have increased in Pakistan from 24 to 45 million from the early 1990s to 2008. Moreover, the World Food Program (WFP) Sustainable Development Policy Research Institute (SDPRI) and Swiss Agency for Development and Cooperation (SADC) claimed that 48.6% of the population of Pakistan lives below the food security line [33]. Of the total food insecure population 22.4 percent are extremely food insecure in the country. In Sindh, the province in which this study was conducted, 44.3% of population has food insecurity [34]. Our research has shown a direct pathway for boys and girls, such that more food insecurity is associated with a greater likelihood of being punished. Food security is essential for social, cognitive and emotional development. Inadequate supply of food to the body has short term effects of irritation, anger and violent behavior (i.e., perpetration) and long term effects of inadequate nutrients can cause irreversible damage to the brain [35]. Food insecurity is rooted in financial insecurity (i.e. poverty). A study among school-aged children in Yemen links poverty and food insecurity to corporal punishment [31]. Malnourished and impoverished children suffer not only from structural violence but their irritable behavior and poor academic performance can result in corporal punishment by parents and school teachers, therefore causing a cycle of continued violence [35]. Our research documents that girls report less hunger compared to boys. Our results support that hunger, violent peer perpetration, and corporal punishment at school is significantly more likely to be reported by boys than girls. Studies from African-American and Egyptian schools confirm these gender differences [28, 36].

Additionally, this study shows indirect pathways between hunger and corporal punishment that are mediated by depression, poor academic performance and days missed from school for girls and depression, poor academic performance, engagement in peer violence and physical punishment at home for boys. These children who are vulnerable due to hunger, depression, beating at home and already have impaired school performance (partly due to missing days) are the most abused by teachers in the form of corporal punishment. The pathway between hunger and depression has been supported by other research and has shown that children can be depressed due to lack of food, and their depression may increase if their mothers are also depressed [35]. It is well recognized that depressed children can be more victimized [37] and is shown in our pathway for boys. Our pathway seen in boys that is mediated by corporal punishment at home, depression and poor school performance closely mirrors that described in work in Tanzania [38]. Scant research exists on the association between poor academic performance and school corporal punishment [31]. However, one study conducted in Pakistan shows a significant association between school corporal punishment and academic performance, attendance, dropout ratio and mental health factors [19]. Finally, a study conducted in Pakistan show school corporal punishment as the main reason for school dropout and deviant behavior among students [39].

### Limitations

All findings are associations. We do not know temporal sequencing and cannot infer causality. Our research methodology has limitations that it may under- or over-represent school and parent corporal punishment, youth-to-youth victimization and perpetration as well as characteristics of family life and youth academic performance. The questions may miss some types and episodes of corporal punishment, youth victimization or perpetration and incorrectly classify others, particularly with respect to the 4- week reporting period. Children may not accurately recall the timing and type of victimization or perpetration they experienced (i.e., whether or not the exposure occurred within the last four weeks). The researchers acknowledge recall bias is potentially operant in all questions. Finally, our participants were limited to Sindhi and Urdu speakers, although these are the languages of teaching in the participating schools. Despite these limitations, the researchers feel this study provides a framework for examining the occurrence of school and parent corporal punishment within the context of youth-to-youth violence and association with youth academic performance as well as related characteristics of family life, such as food security. We feel this research the most detailed and comprehensive data available on the frequency and severity of school and parent corporal punishment and associated youth-to-youth perpetration and victimization for male and female youth in grade 6 in urban public schools in Pakistan.

## Conclusions

Boys, age 11 to 14, are more likely than girls of the same age to experience corporal punishment at school. We have shown that children who are most vulnerable due to food insecurity, depression, being beaten at home and those struggling with performance at school are most likely to be beaten by teachers. All of the paths in the structural models initially stemmed from poverty, which for both boys and girls directly results in beatings. Strategies to eliminate all forms of corporal punishment are urgently needed. However, these findings also call attention to ponder on strategies to maximize food security among all population—especially children.

## Supporting information

S1 File This file contains data collected from 1752 students. (SAV)

S2 File This file contains questionnaire in English language. (DOCX)

S3 File This file contains questionnaire used in local language Urdu. (PDF)

S4 File This file contains questionnaire used in local language Sindhi. (PDF)

## References

[1] Straus MA, editor Differences in corporal punishment by parents in 32 Nations and its relation to national differences in IQ. 14th International Conference On Violence, Abuse And Trauma, San Diego Retrieved from http://pubpages.unh.edu/mas2/Cp98D%20CP; 2009: Citeseer.

[2] Gershoff ET. School corporal punishment in global perspective: prevalence, outcomes, and efforts at intervention. Psychology, health & medicine. 2017;22(sup1):224–39. 10.1080/13548506.2016.1271955 PMC5560991 28064515

[3] Anderson S, Payne MA. Corporal punishment in elementary education: Views of Barbadian schoolchildren. Child abuse & neglect. 1994;18(4):377–86. 8187023 10.1016/0145-2134(94)90040-x

[4] Hecker T, Hermenau K, Isele D, Elbert T. Corporal punishment and children's externalizing problems: A cross-sectional study of Tanzanian primary school aged children. Child Abuse & Neglect. 2014;38(5):884–92. 24360761 10.1016/j.chiabu.2013.11.007

[5] UNICEF. Child disciplinary practices at home: Evidence from a range of low-and middle-income countries. New York: UNICEF 2010.

[6] Gershoff ET. More harm than good: A summary of scientific research on the intended and unintended effects of corporal punishment on children. Law & Contemp Probs. 2010;73:31. PMC8386132 34446972

[7] Turner HA, Muller PA. Long-term effects of child corporal punishment on depressive symptoms in young adults potential moderators and mediators. Journal of Family issues. 2004;25(6):761–82.

[8] Lansford JE, Chang L, Dodge KA, Malone PS, Oburu P, Palmérus K, et al Physical discipline and children's adjustment: Cultural normativeness as a moderator. Child development. 2005;76(6):1234–46. 10.1111/j.1467-8624.2005.00847.x 16274437 PMC2766084

[9] UNICEF. Corporal punishment in schools in South Asia. Kathmandu: UNICEF, 2001.

[10] Gershoff ET. School corporal punishment in global perspective: prevalence, outcomes, and efforts at intervention. Psychology, health & medicine. 2017:1. 10.1080/13548506.2016.1271955 PMC5560991 28064515

[11] Kilimci S. Teachers’ perceptions on corporal punishment as a method of discipline in elementary schools. The Journal of International Social Research. 2009;2(8):242–51.

[12] Tafa EM. Corporal punishment: the brutal face of Botswana's authoritarian schools. Educational Review. 2002;54(1):17–26.

[13] Ahmad I, Said H, Khan F. Effect of corporal punishment on students' motivation and classroom learning. Review of European Studies. 2013;5(4):130.

[14] Pandey S. Corporal punishment in schools. New Front in Educ. 2001;31:347–54.

[15] Andero AA, Stewart A. Issue of corporal punishment: Re-examined. Journal of Instructional Psychology. 2002;29(2):90.

[16] Gershoff ET. Report on physical punishment in the United States: What research tells us about its effects on children: Center for Effective Discipline Columbus, OH; 2008.

[17] Ahmad I, Said H, Awang Z, Yasin MA-M-Z, Hassan Z, Mansur SSS. Effect of self-efficacy on the relationship between corporal punishment and school dropout. Review of European Studies. 2014;6(1):196.

[18] Arif MS, Rafi MS. Effects of Corporal Punishment and Psychological Treatment on Students' Learning and Behavior. Online Submission. 2007;3(2):171–80.

[19] Naz A, Khan W, Daraz U, Hussain M, Khan Q. The impacts of corporal punishment on students’ academic performance/career and personality development up-to secondary level education in Khyber Pakhtunkhwa Pakistan. International Journal of Business and Social Science. 2011;2(12).

[20] WhatWorks. What Works to Prevent Violence? A Global Programme to Prevent Violence Against Women and Girls. 2015.

[21] Right to Play 2017 [February 9, 2017]. http://www.righttoplay.com/.

[22] McFarlane J, Karmaliani R, Khuwaja HMA, Gulzar S, Somani R, Ali T, et al Preventing Violence Against Children: Methods and Baseline Data of a Cluster Randomized Controlled Trial in Pakistan. Global Health: Science and Practice Journal. 2017 10.9745/GHSP-D-16-00215 28351880 PMC5478222

[23] Mynard H, Joseph S. Development of the multidimensional peer-victimization scale. Aggressive behavior. 2000;26(2):169–78.

[24] Hillis S, Mercy J, Saul J, Gleckel J, Abad N, Kress H. THRIVES: using the best evidence to prevent violence against children. Journal of public health policy. 2016;37(1):51–65. 10.1057/s41271-016-0003-6 27638242

[25] Kovacs M. Children's Depression Inventoryy 2nd Edition (CDI 2)Technical manual. Multi-Health Systems 2011.

[26] Bhatla N, Achyut P, Khan N, Walia S. Are Schools Safe and Gender Equal Spaces? 2015.

[27] Gershoff ET. Corporal punishment by parents and associated child behaviors and experiences: a meta-analytic and theoretical review. Psychological bulletin. 2002;128(4):539 12081081 10.1037/0033-2909.128.4.539

[28] Youssef RM, Attia MS-E-D, Kamel MI. Children experiencing violence II: Prevalence and determinants of corporal punishment in schools. Child Abuse & Neglect. 1998;22(10):975–85. 9793720 10.1016/s0145-2134(98)00084-2

[29] Blain-Arcaro C, Vaillancourt T. Longitudinal Associations between Depression and Aggression in Children and Adolescents. Journal of abnormal child psychology. 2016 Epub 2016/09/28. 10.1007/s10802-016-0204-2 . 27671705

[30] Asad N, Karmaliani R, McFarlane J, Bhamani SS, Somani Y, Chirwa E, et al The intersection of adolescent depression and peer violence: baseline results from a randomized controlled trial of 1752 youth in Pakistan. Child and Adolescent Mental Health. 2017;22(4):232–41. 10.1111/camh.12249 32680419

[31] Alyahri A, Goodman R. Harsh corporal punishment of Yemeni children: occurrence, type and associations. Child abuse & neglect. 2008;32(8):766–73. 18657859 10.1016/j.chiabu.2008.01.001

[32] Griggs D, Stafford-Smith M, Gaffney O, Rockström J, Öhman MC, Shyamsundar P, et al Policy: Sustainable development goals for people and planet. Nature. 2013;495(7441):305–7. 10.1038/495305a 23518546

[33] Mahmood Z, Iftikhar S, Saboor A, Khan AU, Khan M. Agriculture land resources and food security nexus in Punjab, Pakistan: an empirical ascertainment. Food and Agricultural Immunology. 2016;27(1):52–71.

[34] Suleri AQ, Haq S. Food insecurity in Pakistan. 2009.

[35] Chilton M, Chyatte M, Breaux J. The negative effects of poverty & food insecurity on child development. Indian Journal of Medical Research. 2007;126(4):262 18032801

[36] Skiba RJ, Michael RS, Nardo AC, Peterson RL. The color of discipline: Sources of racial and gender disproportionality in school punishment. The urban review. 2002;34(4):317–42.

[37] Marsh HW, Craven RG, Parker PD, Parada RH, Guo J, Dicke T, et al Temporal ordering effects of adolescent depression, relational aggression, and victimization over six waves: Fully latent reciprocal effects models. Dev Psychol. 2016;52(12):1994–2009. Epub 2016/11/29. 10.1037/dev0000241 . 27893244

[38] Hecker T, Hermenau K, Salmen C, Teicher M, Elbert T. Harsh discipline relates to internalizing problems and cognitive functioning: findings from a cross-sectional study with school children in Tanzania. BMC Psychiatry. 2016;16:118 Epub 2016/05/01. 10.1186/s12888-016-0828-3 . 27129400 PMC4850652

[39] Chitrali JA, Anwar M, Nabahat S. Dynamics of Gender Based Violence: Investigating the Effects of Violence in Pakistani School on School Dropout and Loss of Creativity among Students. Pakistan Journal of Criminology. 2014;6(2):133.

